# Piezo1 induces mitochondrial autophagy dysfunction leading to cartilage injury in knee osteoarthritis

**DOI:** 10.1186/s10020-025-01335-x

**Published:** 2025-08-02

**Authors:** Likai Yu, Zishan Su, Di Tian, Shangqi Liu, Li Zhang, Zeen Wang, Shaobo Guo, Wenhui Zhu, Peimin Wang, Nongshan Zhang

**Affiliations:** 1 Department of Orthopedics, Jiangsu Province Hospital of Chinese Medicine, Nanjing, Jiangsu 210029 China; 2 First College of Clinical Medicine, Nanjing University of Chinese Medicine, Nanjing, Jiangsu 210023 China; 3 Orthopedics of traditional Chinese medicine, Zhongda Hospital Southeast University, Nanjing, Jiangsu 210009 China

**Keywords:** Piezo1, Cartilage, Mitochondria, Autophagy, Knee osteoarthritis

## Abstract

**Background:**

External mechanical stress plays a pivotal role in the pathogenesis of knee osteoarthritis. Piezo1 can sense mechanical stress changes on the surface of various cell types and convert them into bioelectrical signals to regulate cellular functions. Therefore, our study aimed to investigate the role of Piezo1 in mechanically induced KOA and elucidate its underlying mechanisms.

**Methods:**

In this study, we employed various techniques to assess the effects of mechanical stress on knee joint cartilage in vivo and in vitro experiments. In vivo, we performed Micro-CT scanning, H&E staining, and ELISA analysis on the knee joints to evaluate the degree of cartilage damage and the expression of pro-inflammatory factors. In vitro, we utilized a cell stretcher to apply mechanical stress specifically to chondrocytes. Subsequently, we investigated the expression levels of Piezo1, pro-inflammatory factors, Collagen II, and other relevant markers within the chondrocytes. This approach aimed to shed light on the potential impact of Piezo1 on chondrocytes when subjected to mechanical stress.

**Results:**

Elevated expression of Piezo1 was observed in the cartilage of mice post-treadmill exercise intervention, with noticeable damage to the cartilage tissue and reduced surface smoothness. External mechanical stress significantly lowered the synthesis of the extracellular matrix in chondrocytes, potentially through the inhibition of mitochondrial autophagy levels, leading to increased mitochondrial dysfunction and the induction of pro-apoptotic proteins and pro-inflammatory cytokines.

**Conclusions:**

Mechanical stress induces extracellular matrix degradation and promotes KOA progression through Piezo1-mediated chondrocyte autophagy dysfunction and apoptotic injury.

**Supplementary Information:**

The online version contains supplementary material available at 10.1186/s10020-025-01335-x.

## Introduction

Osteoarthritis (OA) is a chronic degenerative disease that affects multiple joints, primarily characterized by the progressive deterioration of articular cartilage, as well as pathological changes in subchondral bone and surrounding synovial structures (Hunter and Bierma-Zeinstra 2019). Clinically, OA commonly occurs in joints such as the knee, hip, and those of the hands and feet, with the highest incidence observed in the knee joint. Symptoms include knee pain, swelling, and functional impairment(Jang et al. 2021). Throughout the progression of knee osteoarthritis (KOA), cartilage damage is widely recognized as one of the most critical pathological alterations, involving mechanisms such as chondrocyte apoptosis, extracellular matrix (ECM) degradation, autophagy dysregulation, and mitochondrial dysfunction (Giorgino et al. 2023). Excessive apoptosis often induces the overexpression of catabolic enzymes and a decline in anabolic processes, further leading to tissue damage (Jiang et al. 2020). In contrast, numerous studies focusing on cellular states have demonstrated that autophagy—a common and highly conserved cellular recycling process—can degrade unnecessary cellular components into fundamental nutrients such as amino acids, lipids, and sugars via lysosomes, playing a protective role in preventing cartilage degeneration during KOA progression (Zhang et al. 2021). Research by Almonte-Becerril et al. demonstrated that during the early stages of KOA, the expression of microtubule-associated protein 1 light chain 3 (LC3) in chondrocytes increases, and autophagy is activated as an adaptive response to prevent excessive cell death (Almonte-Becerril et al. 2010). As the disease progresses, caspase signaling associated with apoptosis shuts down the autophagy-lysosome process, switching the protective autophagy program to apoptosis, at which point severe cartilage damage occurs (Yang et al. 2020).

In addition to genetic and metabolic factors, abnormal mechanical stress stimulation is also a significant factor capable of regulating chondrocyte states and influencing KOA progression(Sun et al. 2021a, b). Studies indicate that excessive mechanical stress can upregulate the expression of mechanosensitive ion channel Piezo1 in chondrocytes, thereby increasing chondrocyte apoptosis and leading to cartilage degeneration, although the precise mechanisms remain incompletely understood (Qin et al. 2021). Piezo1 is a mechanosensitive cationic-gated channel responsible for sensing and transducing external mechanical stress into intracellular calcium influx, thereby regulating physiological and pathological processes including cell proliferation, senescence, differentiation, and death (Cox et al. 2016). In 2014, Lee et al. first demonstrated the expression of Piezo1 in human articular cartilage and found that using the mechanosensitive channel inhibitor GsMTx4 could significantly reduce Piezo1 activation-induced chondrocyte death (Lee et al. 2014). Building upon this, studies have demonstrated that under stimulation from a rigid extracellular environment, Piezo1 becomes significantly activated and induces reactive oxygen species (ROS) release, thereby triggering oxidative stress responses and nucleus pulposus cell apoptosis (B. Wang et al. 2021a, b). Furthermore, researchers have found that applying high-intensity mechanical forces to nucleus pulposus cells leads to increased Piezo1 expression, accompanied by decreased levels of autophagy-related markers such as LC3 and Beclin-1 (Shi et al. 2022). This suggests that Piezo1 induces cellular autophagy inhibition in response to mechanical stress stimulation and may play a role in cartilage damage associated with KOA.

Notably, Piezo1-mediated calcium influx disrupts intracellular calcium homeostasis, subsequently impairing mitochondrial function, primarily manifested by changes in membrane potential and significant ROS release (Tsai et al. 2016). Mitophagy serves as a crucial mechanism for mitochondrial quality control by eliminating damaged mitochondria and excess ROS, thereby maintaining mitochondrial function and preventing further cell death (Lu et al. 2023). Similarly, autophagy activation regulates oxidative stress by recycling damaged cellular components (such as the endoplasmic reticulum and peroxisomes), thereby promoting cell survival (Su et al. 2023). Studies have shown that autophagy and mitophagy play significant roles in various disease processes, including osteoarthritis, cardiovascular diseases, and neurological disorders (Bravo-San Pedro et al. 2017; Fang et al. 2019; K. Sun et al. 2021a, b). However, to date, the relationship between Piezo1 expression and cellular autophagy in KOA cartilage tissue remains unexplored. Therefore, this study aims to investigate whether the Piezo1 channel in knee joint cartilage tissue can sense mechanical stress and influence cellular autophagy and apoptosis processes, thereby contributing to the progression of KOA.

## Materials and methods

### Animal grouping and interventions

This study involved two separate batches of experimental animals. All mice were purchased from Beijing Vital River Laboratory Animal Technology Co., Ltd. (Beijing, CHN). To avoid interference from age and gender differences in constructing the KOA animal model, this study utilized adult male mice for the experiments. The first batch consisted of 30 male C57BL/6 mice aged 8 weeks. After one week of acclimatization feeding, they were randomly divided into a Control group (*n* = 15) and a Treadmill group (*n* = 15). The Treadmill group was subjected to excessive mechanical stress through treadmill exercise. Following the acclimatization period, the mice underwent an adaptation training for 3 days at a speed of 10 m/min, twice daily, for 15 min each session. Upon completion of the adaptation training, formal training commenced with the treadmill set at a speed of 25 m/min and an incline of 15°, for 1 h per day, 6 days a week, for a duration of 8 weeks (Li et al. 2017).

The second batch included 45 male C57BL/6 mice aged 8 weeks. After one week of acclimatization feeding, they were randomly allocated to a Control group (*n* = 15), a Treadmill group (*n* = 15), and a Treadmill + GsMTx4 group (*n* = 15). For the Treadmill + GsMTx4 group, starting from the 4th to the 8th week after intervention, a daily intraperitoneal injection of 1 mg/kg GsMTx4 (MCE, USA) was administered (Wang et al. 2021a, b). The Control group was maintained on regular feeding without any interventions. After the intervention period, euthanasia was performed on all mice, and their knee joints were dissected and processed.

All animals were treated in accordance with the principles of the Declaration of Helsinki and ARRIVE guidelines, and all protocols were approved by the Experimental Animal Management Committee and the Animal Ethics Committee of Nanjing University of Chinese Medicine(approval number: 202308A002).

### Micro-CT examination

Following the completion of the animal intervention, knee joints of the mice were examined using Micro-CT (Quantum GX, USA), and the acquired images and data were processed and analyzed using computer software to assess the degree of cartilage damage in each group.

Micro-CT Scanner Parameters: The scanner was set to a voltage of 50 kV and a current of 200µA, with a total rotation angle of 360° and an angular increment of 0.4°, and a layer thickness of 9 μm. After scanning, the two-dimensional image sequences were automatically reconstructed into three-dimensional volumes with isotropic voxel sizes of 15.9 μm using Skyscan NRecon software and the Feldkamp cone-beam reconstruction algorithm.

### Histopathology

The harvested murine cartilage tissues were fixed in 4% perfluoroalkoxy (PFA) at room temperature for 24 h, embedded in paraffin, and sectioned into 4 μm slices. The sections were stained using the Hematoxylin-Eosin (H&E) Stain Kit (Solarbio, CHN), the Modified Saffron-O and Fast Green Stain Kit (for Bone) (Solarbio, CHN), and the Alcian Blue Periodic Acid Schiff (AB-PAS) Stain Kit (Solarbio, CHN), followed by encapsulation with resin mounting medium for preservation and examination.

### Enzyme linked immunosorbent assay (ELISA)

Concentrations of Interleukin-1β (IL-1β) and tumor necrosis factor-α (TNF-α) in the serum or chondrocyte supernatants from different groups of mice were detected using ELISA kits (mlbio, CHN). Procedures were performed according to the manufacturer’s protocols, and the corresponding OD values were measured with a microplate reader (PerkinElmer, USA).

### Western blotting (WB)

Proteins were extracted from murine chondrocytes using RIPA lysis buffer containing 1 mM PMSF, and the protein concentrations were measured with a BCA Protein Assay Kit (Beyotime, China). The proteins from each group were separated by SDS-PAGE and then transferred onto 0.45 μm PVDF membranes (Millipore, USA). After blocking with QuickBlock™ Western Blocking Buffer (Beyotime, China) for 1 h, the membranes were incubated overnight at 4 °C with primary antibodies against β-actin, Piezo1, Collagen II, Aggrecan, Matrix metalloproteinases-13 (MMP-13), Recombinant A Disintegrin and Metalloproteinase with Thrombospondin 5 (ADAMTS-5), LC3A/B, p62, B-cell lymphoma-2 (BCL2), BCL-2-associated X protein (Bax), Beclin-1, cleaved caspase-3 (CC3), PTEN induced putative kinase 1 (PINK1), Parkin, and BCL2 interacting protein 3 (BNIP3) in a solution. Subsequently, the membranes were incubated with the appropriate secondary antibodies. Bands were detected using ECL detection reagents (Vazyme, China). Protein band densities were quantified using Image J software version 1.53. The specific details of the antibodies, including manufacturers, catalog numbers, and dilution ratios, are listed in Table 1.

**Table 1:** Antibody related parameters

Antibody	Reagent dealer	Dilution ratio
β-actin	CST; 4967 L	WB: 1:1000
Piezo1	Abacm; ab128245	WB: 1:1000
	Affinity; DF12083	IF: 1:100
		IHC: 1:300
Collagen II	Affinity; AF0135	WB: 1:1500
		IHC: 1:200
Aggrecan	SantaCruz; sc-166,951	WB: 1:500
MMP-13	CST; 69,926	WB: 1:1000
ADAMTS-5	Abacm; ab41037	WB: 1:250
LC3A/B	CST; 4108 S	WB: 1:1000
LC3B	Affinity; AF4650	IF: 1:100
p62	CST; 39,749 S	WB: 1:1000
	Affinity; AF5384	IF: 1:300
BAX	CST; 2772	WB: 1:1000
BcL2	CST; 3498	WB: 1:1000
Beclin-1	Abacm; ab210498	WB: 1:1000
Cleaved-Caspase3	CST; 9661T	WB: 1:1000
PINK1	Abacm; ab186303	WB: 1:1000
Parkin	Abacm; ab77924	WB: 1:2000
BNIP3	Abacm; ab109362	WB: 1:1000

### Real-time quantitative PCR (RT-qPCR) analysis

Total RNA was extracted from murine cartilage tissues or chondrocytes using TRIzol reagent (Vazyme, China). The extracted total RNA was reverse-transcribed to cDNA using HiScript III All-in-one RT SuperMix Perfect for qPCR (Vazyme, China). RT-qPCR was performed with ChamQ SYBR qPCR Master Mix (Vazyme, China) in a reaction system comprising 10 µl of 2× ChamQ SYBR qPCR Master Mix, 0.4 µl of each primer, and 9.2 µl of diluted cDNA. Amplification conditions were set as follows: initial denaturation at 95 °C for 30 s followed by 40 cycles at 95 °C for 10 s and 60 °C for 30 s. Relative gene expression was determined using the 2^−ΔΔCt^ method and normalized to the levels of β-actin. Details of the primer designs are provided in Table 2.

**Table 2:** Primer design sequence|

Target gene	Sequence(5’−3’)
β-actin	F: GTACTCTGTGTGGATCGGTGG
	R: AACGCAGCTCAGTAACAGTCC
Piezo1	F: CTTCGGGTTGGAGAGGTACG
	R: ACTCAAAGGCTCTTCGGCTC
Collagen II	F: CAAGGACCCAGAGGTGATCG
	R: CCAGCCTTCTCGTCATACCC
Aggrecan	F: GATGGAAACCAGCACGGAGA
	R: CCTGGGAAACAGTGGGTTCA
MMP-13	F: TACCATCCTGCGACTCTTGC
	R: TTCACCCACATCAGGCACTC
ADAMTS-5	F: CCAAGGCCAAATGGTGTGTC
	R: CAATGGCGGTAGGCAAACTG
LC3II	F: TTGTCATCGTGGGAACTGGG
	R: GGTGGCAAGGTATCGACCAA
p62	F: GAGTCCCTCTCCCAGATGCT
	R: GCCAAGACACTGGGCCTATC
Bax	F: CAGGATCGAGCAGGGAGGAT
	R: CAGCTTCTTGGTGGACGCAT
BcL2	F: GAGGCAGGCGATGAGTTTGA
	R: CACGATGCGACCCCAGTTTA
Beclin-1	F: TAGCTGAAGACCGGGCGAT
	R: CCACCCAGGCTCGTTCTAC
PINK1	F: GGCCAGACAGGGAATGAAGT
	R: TATGAGCCATGCTGGTTGCT
Parkin	F: CCTGCAAACAAGCAACCCTC
	R: CACCACTCATCCGGTTTGGA
BNIP3	F: GCGTGCGGGTTATCTGTAAA
	R: GTGGACAGCAAGGCGAGAAT

### RNA sequencing

Total RNA was extracted from cartilage of the Control group and Treadmill group mice using the TRIzol reagent. The RNA was then purified and its integrity assessed by LC-Bio Technologies (Hangzhou) Co., Ltd. (Hangzhou, CHN) for RNA sequencing. Subsequent Principal Components Analysis (PCA) and generation of corresponding heatmaps were performed on the OmicStudio cloud platform (https://www.omicstudio.cn). Kyoto Encyclopedia of Genes and Genomes (KEGG) analysis were conducted, with the respective bar charts being generated.

### Primary chondrocyte extraction and culture

Primary chondrocytes were extracted and cultured from 1-week-old male C57BL/6 mice (Jiangsu Huachuang Sino Pharmatech Co., Ltd.) following euthanasia via intraperitoneal injection of an overdose of sodium pentobarbital. In a sterile environment, the skin surrounding the mouse knee joints was incised, the muscles around the joint were dissected, and the cartilage was extracted. The cartilage tissue was then minced and placed in DMEM high-glucose medium (Gibco, USA) containing 4 mg/ml type II collagenase (Solarbio, China), and digested at 37 °C for 6 h. After digestion, residual tissue was filtered through a 70 μm cell strainer (Biosharp, China) and transferred to a 15 ml conical centrifuge tube for cell collection. Cells were cultured in DMEM high-glucose medium supplemented with 10% FBS and 1% p/s solution (complete culture medium) at 37 °C in a 5% CO_2_ incubator. The culture medium was replaced every two days. Cells from the third passage were suitable for subsequent experiments.

### Tensile strain cell model

Chondrocytes in the logarithmic growth phase with good viability were selected for the tensile strain model. Cells were digested with 0.25% trypsin, pipetted to disperse clumps, and then transferred into a 15 ml centrifuge tube for centrifugation. The supernatant was discarded, cells were resuspended in complete culture medium, and seeded into 6-well BioFlex culture plates to be cultured for 24 h before subsequent experiments. For the mechanical stress-loading group, a Flexcell FX5000 Tension system (Flexcell International, USA) was employed to apply mechanical tensile stresses. The parameters were as follows: frequency of 1.0 Hz, and tensile magnitudes of 5%, 10%, 15%, and 20% over durations of 6 h, 12 h, 24 h, and 48 h (Rendon et al. 2022). Control groups were cultured under identical conditions without the application of mechanical stress. When employing the Piezo1 inhibitor GsMTx4 in chondrocytes, cells were pre-treated with 5 µM GsMTx4 prior to the application of mechanical stress (1.0 Hz, 10% tensile magnitude, for 12 h).

### Immunofluorescence

Post-intervention chondrocytes were fixed using 4% PFA, permeabilized for 10 min at room temperature with Enhanced Immunostaining Permeabilization Buffer (Beyotime, China), and subsequently blocked for 90 min with Immunol Staining Blocking Buffer (Beyotime, China). Following treatment, cells were incubated overnight at 4 °C with primary antibodies against Piezo1, LC3B, and p62. After primary incubation, the cells were incubated with secondary antibodies for 2 h at room temperature protected from light. Finally, cell nuclei were stained for 10 min with Antifade Mounting Medium containing DAPI. After the aforementioned treatments, an Inverted fluorescence microscope (Nikon, JPN) was used for observation, and fluorescence intensity was measured using Image J software version 1.5.3. Details of the antibodies including manufacturers, catalog numbers, and dilution ratios are listed in Table 1.

### Mitochondrial membrane potential (ΔΨm) assay

After the chondrocyte intervention was completed, the cells were treated using an Enhanced Mitochondrial Membrane Potential Assay Kit with JC-1 (Beyotime, China) following the instructions provided with the kit. Upon completion of the treatment, the ratio of positive cells was detected using Flow Cytometry (Beckman Coulter CytoFLEX).

### ROS detection

Detection was conducted using Dihydroethidium (DHE) (MCE, USA). Chondrocytes were seeded into 6-well plates, and following intervention, the cells were incubated for 20 min in serum-free medium containing 5 µM DHE. The culture medium was then discarded, and the cells were washed three times with PBS. Subsequently, cells were observed using an Inverted Fluorescence Microscope (Nikon, JPN).

### Mito-SOX red staining

Mitochondrial superoxide was assessed using Mito-SOX Red (MCE, USA). Chondrocytes were plated in 6-well plates and post-intervention, the cells were incubated for 30 min in serum-free medium supplemented with 1 µM Mito-SOX Red. Following incubation, the medium was removed and the cells were washed three times with PBS. Observation of cells was then performed using an Inverted Fluorescence Microscope (Nikon, JPN).

### Mito-tracker red staining

Mitochondrial mass in live cells was measured using Mito-Tracker Red CMXRos (Beyotime, CHN). Chondrocytes were seeded into 6-well plates and following the intervention, were incubated for 20 min in serum-free medium containing 100 nM Mito-Tracker Red CMXRos. The medium was then aspirated and the cells were washed three times with PBS. Subsequently, observations were made using an Inverted Fluorescence Microscope (Nikon, JPN).

### Cell apoptosis detection

Apoptosis in chondrocytes was assessed using the Annexin V-FITC/PI Apoptosis Detection Kit (MCE, USA). Chondrocytes were seeded in 6-well plates, and following intervention, the cells were collected and gently resuspended in 195 µL of Binding Buffer. Subsequently, 5 µL of Annexin V-FITC and 10 µL of PI Stain were added and the mixture was gently vortexed. This was followed by incubation for 15 min at room temperature, protected from light. After the incubation, the ratio of positive cells was analyzed using Flow Cytometry (BD FACSCelesta).

### Transmission Electron Microscopy (TEM)

Autophagy in chondrocytes was observed using transmission electron microscopy. Chondrocytes were seeded onto 6-well plates, and after the intervention, they were preserved in electron microscopy fixative. Transmission electron microscopy was then used to directly observe the morphological changes of mitochondria and autophagosomes in chondrocytes following different interventions.

### TUNEL staining

TUNEL staining was conducted to detect nuclear DNA fragmentation during apoptosis. Following the manufacturer’s instructions of the One Step TUNEL Apoptosis Detection Kit (MCE, China), chondrocyte tissue or cells were fixed and permeabilized before incubation with 50 µL of TUNEL reaction mixture at 37 °C in the dark for 1 h. This was followed by three washes with PBS. Finally, the slides were mounted using Antifade Mounting Medium with DAPI and examined under an Inverted Fluorescence Microscope (Nikon, JPN) for fluorescence assessment.

### Immunohistochemical (IHC)

Chondrocyte tissue paraffin blocks were sectioned into 5 μm slices, which were then dried after retrieving to prevent detachment and baked for 2 h. Subsequent to baking, the sections underwent deparaffinization, hydration, antigen retrieval, and blocking steps before the application of primary antibodies against Piezo1, Collagen II, etc. The sections were then incubated overnight at 4 °C in a specialized incubation box. Following this, the sections were incubated with the secondary antibody for 1 h. DAB (3,3’-diaminobenzidine) reagent was used for color development, and finally, hematoxylin was applied for nuclear counterstaining. Immunohistochemical analysis was performed by quantifying the integrated optical density (IOD) per area using ImageJ software to evaluate marker expression levels. Details of the antibody manufacturers, catalog numbers, and dilution ratios are provided in Table 1.

### Statistical analysis

All experiments were performed with at least three biological replicates. All statistical analyses were conducted using Graphpad Prism 9.5. Data are presented as mean ± standard deviation (SD). All data met the assumptions of normal distribution. Statistical evaluations were carried out using t-tests (two-tailed) or one-way analysis of variance (ANOVA) with Tukey’s post hoc test. A *p* < 0.05 was considered to indicate statistical significance.

## Results

### Mechanical stress overload induces mouse knee cartilage injury

**Fig. 1:**
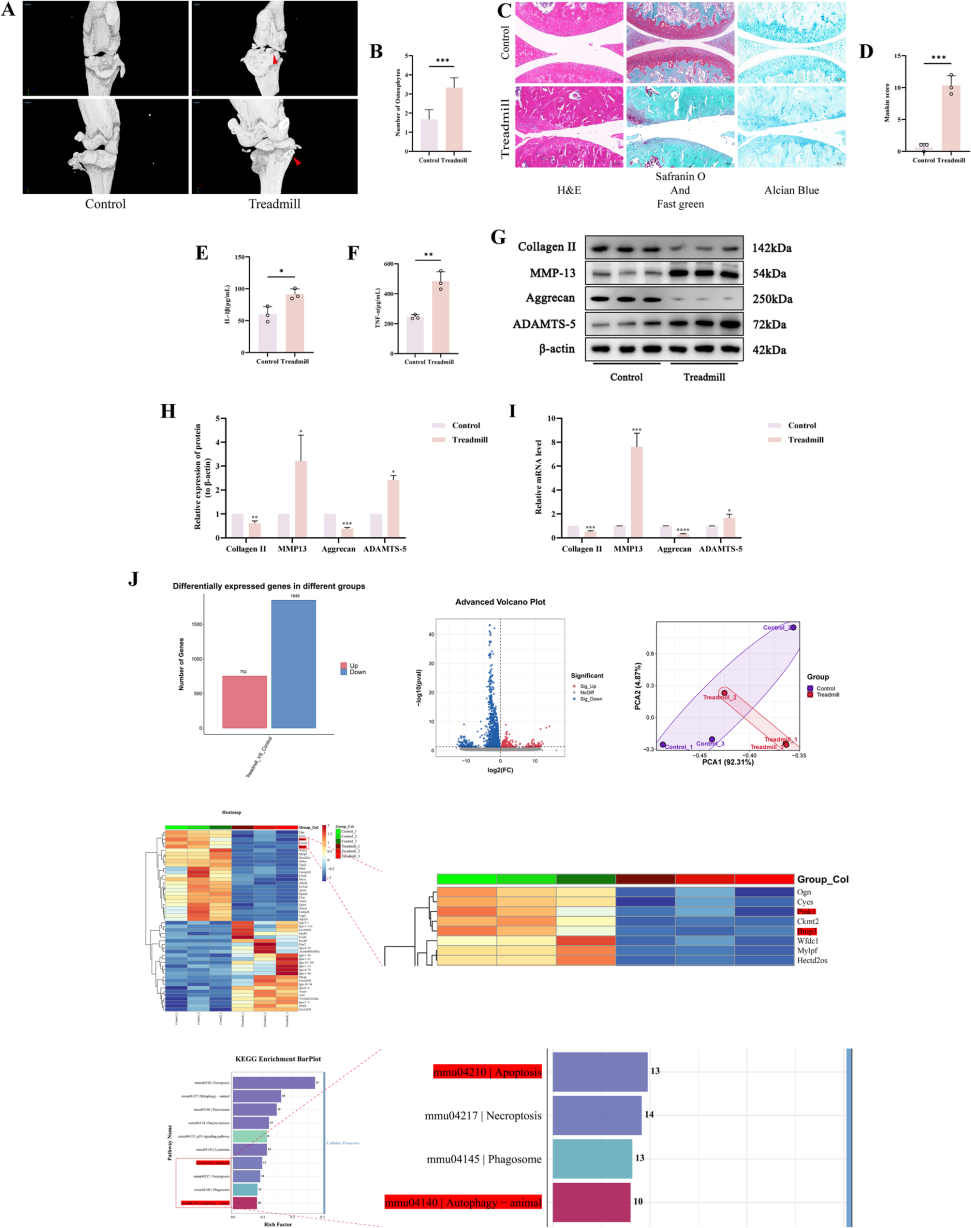
Mechanical Stress Overload Induces Mouse Knee Cartilage Injury. **A** Micro CT images of knee joints of mice. Arrows show the formation of osteophytes. **B** Osteophyte number assay based on Micro-CT. **C** Images of mouse cartilage stained with H&E, Safranin O-Fast Green and Alcian Blue. (Scale bar 100 μm). **D** Mankin score of mouse knee joint. **E**,** F** Detection of IL-1β and TNF-α levels in mouse peripheral blood serum by ELISA. **G**,** H** Western blot bands represent the expression levels of Collagen II, MMP-13, Aggrecan and ADAMTS-5 proteins in different groups of mouse cartilage, with histograms representing the quantitative data of each index standardized to β-actin. **I** Detection of Collagen II, MMP-13, Aggrecan and ADAMTS-5 mRNA levels in mouse cartilage by RT-qPCR. **J** Data visualization analysis of RNA seq Values are expressed as the mean ± SD from three independent experiments (*n* = 3). *, **, *** and **** indicate *p* < 0.05, *p* < 0.01, *p* < 0.001 and *p* < 0.0001, respectively

To verify that mechanical stress overload can cause cartilage injury in the knee joints of C57BL/6 mice, we randomly assigned C57BL/6 mice into a Control group and a Treadmill group. Following the intervention, a series of experimental studies were carried out. Micro-CT analysis revealed an increased number of osteophytes in the knee joints of the Treadmill group mice compared to the control group (*p* < 0.05) (Fig. 1A, B). Subsequent histopathological staining of the cartilage tissue with H&E, Saffron O and Fast Green, and Alcian Blue indicated prominent wear and rupture in the Treadmill group in comparison with the Control group, with a significant increase in the Mankin score (*p* < 0.05) (Fig. 1C, D). ELISA assays for mouse serum IL-1β and TNF-α levels suggested greater release of pro-inflammatory factors in the Treadmill group as opposed to the Control group (*p* < 0.05) (Fig. 1E, F). WB analysis for markers of cartilage injury showed a huge decrease in the protein expression of Collagen II and Aggrecan, and a significant increase in MMP-13 and ADAMTS-5 in the Treadmill group as opposed to the Control group (*p* < 0.05) (Fig. 1G, H). RT-qPCR analysis for mRNA expression of the aforementioned markers also displayed a similar trend (*p* < 0.05) (Fig. 1I).

To further elucidate the potential mechanisms by which mechanical stress overload causes cartilage injury in mice, RNA-seq was performed on cartilage tissues from both groups. The results indicated that in comparison to the Control group, the Treadmill group had 2600 differentially expressed genes (with 752 upregulated and 1848 downregulated genes). Notably, we found a significant downregulation in the gene expression of PINK1 and BNIP3, and KEGG enrichment analysis suggested that the related genes were enriched in pathways involved in autophagy and apoptosis (Fig. 1J). Thus, we hypothesize that cartilage injury caused by mechanical stress overload may be closely associated with cell apoptosis and mitochondrial autophagy.

### Impact of mechanical stress on the expression of Piezo1 in knee joint cartilage

**Fig. 2:**
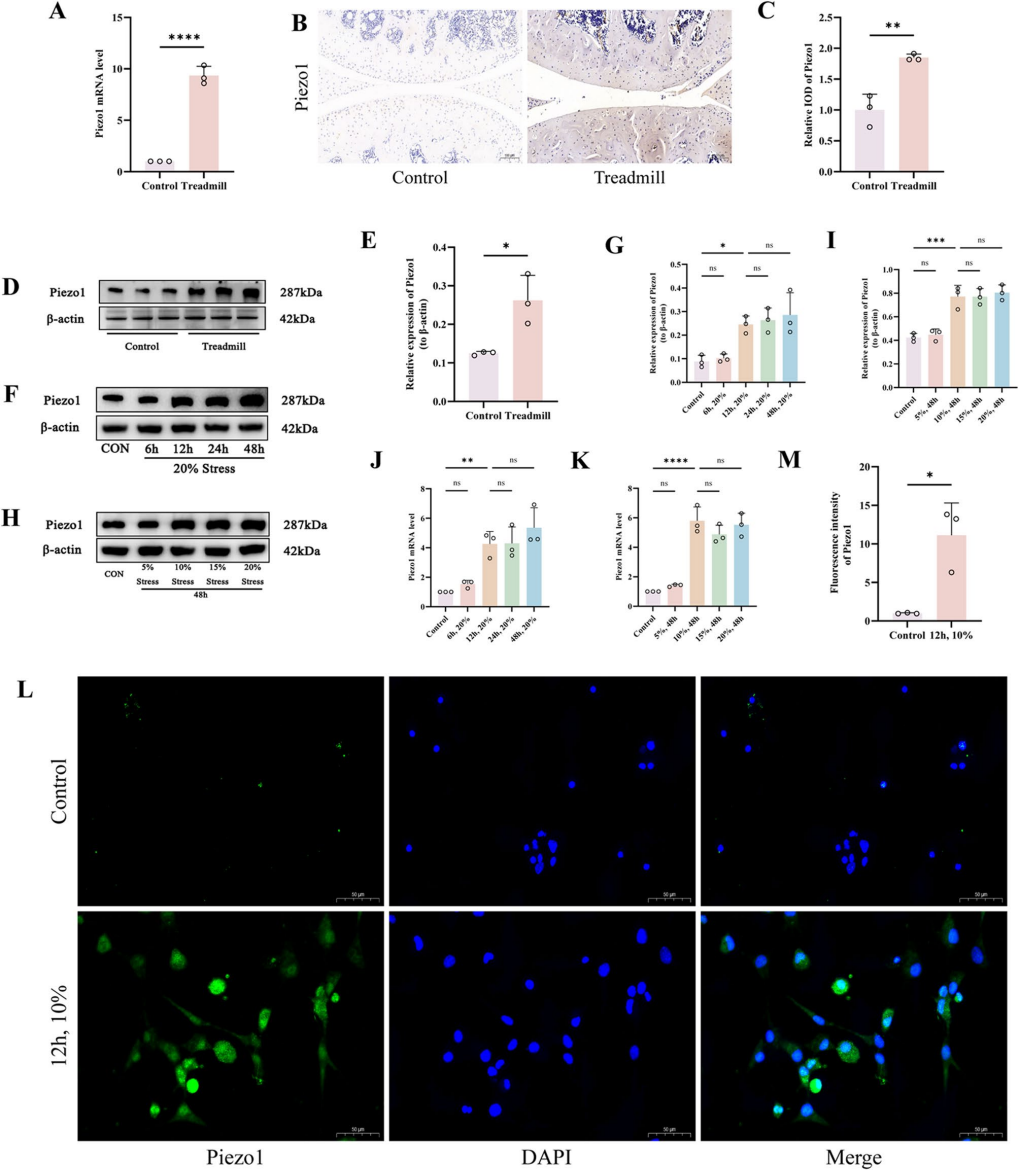
Impact of Mechanical Stress on the Expression of Piezo1 in Knee Joint Cartilage. **A** Detection of Piezo1 mRNA levels in mouse cartilage by RT-qPCR. **B**,** C** Piezo1 immunohistochemistry images of mouse cattilage tissue. (Scale bar 100 μm). **D**,** E** Western blot bands represent the expression levels of Piezo1 proteins in different groups of mouse cartilage, with histograms representing the quantitative data of each index standardized to β-actin. **F-I** Western blot bands represent the expression levels of Piezo1 proteins in different groups of cartilage cells, with histograms representing the quantitative data of each index standardized to β-actin. **J**,** K** Detection of Piezo1 mRNA levels in different groups of cartilage cells by RT-qPCR. **L**,** M** Immunofluorescence detection of Piezo1 expression in different groups of cartilage cells, accompanied by quantitative histograms. (Scale bar 50 μm) Values are expressed as the mean ± SD from three independent experiments (*n* = 3). *, **, *** and **** indicate *p* < 0.05, *p* < 0.01, *p* < 0.001 and *p* < 0.0001, respectively

To confirm that mechanical stress can regulate the expression of Piezo1 in cartilage, we conducted both in vivo and in vitro experiments for validation. Initially, in our in vivo study, RT-qPCR revealed a significant upregulation of Piezo1 mRNA expression in the Treadmill group as opposed to the Control group (*p* < 0.05) (Fig. 2A). Concurrently, IHC showed that the expression of Piezo1 was considerably greater in the Treadmill group than in the Control group (*p* < 0.05) (Fig. 2B, C). WB also found that the protein expression of piezo1 in treadmill group was far greater than that in control group (*p* < 0.05) (Fig. 2D, E). Subsequently, our in vitro experiments varied the intensity of tensile stress or the duration of intervention to clarify the effect of mechanical stress on Piezo1 in chondrocytes. We discovered that under a constant tensile force, the protein and mRNA expression of Piezo1 in all groups were significantly higher than those in the Control group after 12 h of intervention (*p* < 0.05) (Fig. 2F, G, J). When the duration of the tensile force was constant, an intensity of 10% resulted in a huge increase in Piezo1 protein and mRNA expression in all groups as opposed to the Control group (*p* < 0.05) (Fig. 2H, I, K). Therefore, the cell tensile stress intervention parameters for subsequent experiments were set as follows: frequency at 1.0 Hz, tensile strength at 10%, and duration of intervention at 12 h. Lastly, upon intervening with chondrocytes under these conditions, we observed that the Piezo1 the intensity of fluorescence in the 12 h, 10% group was much greater than that in the Control group (*p* < 0.05) (Fig. 2L, M). Hence, we have determined that mechanical stress can affect the expression of Piezo1 in cartilage and established the corresponding intervention parameters for in vitro experiments.

### Mechanical stress overload leads to chondrocyte mitochondrial dysfunction, apoptosis, and autophagy inhibition

**Fig. 3:**
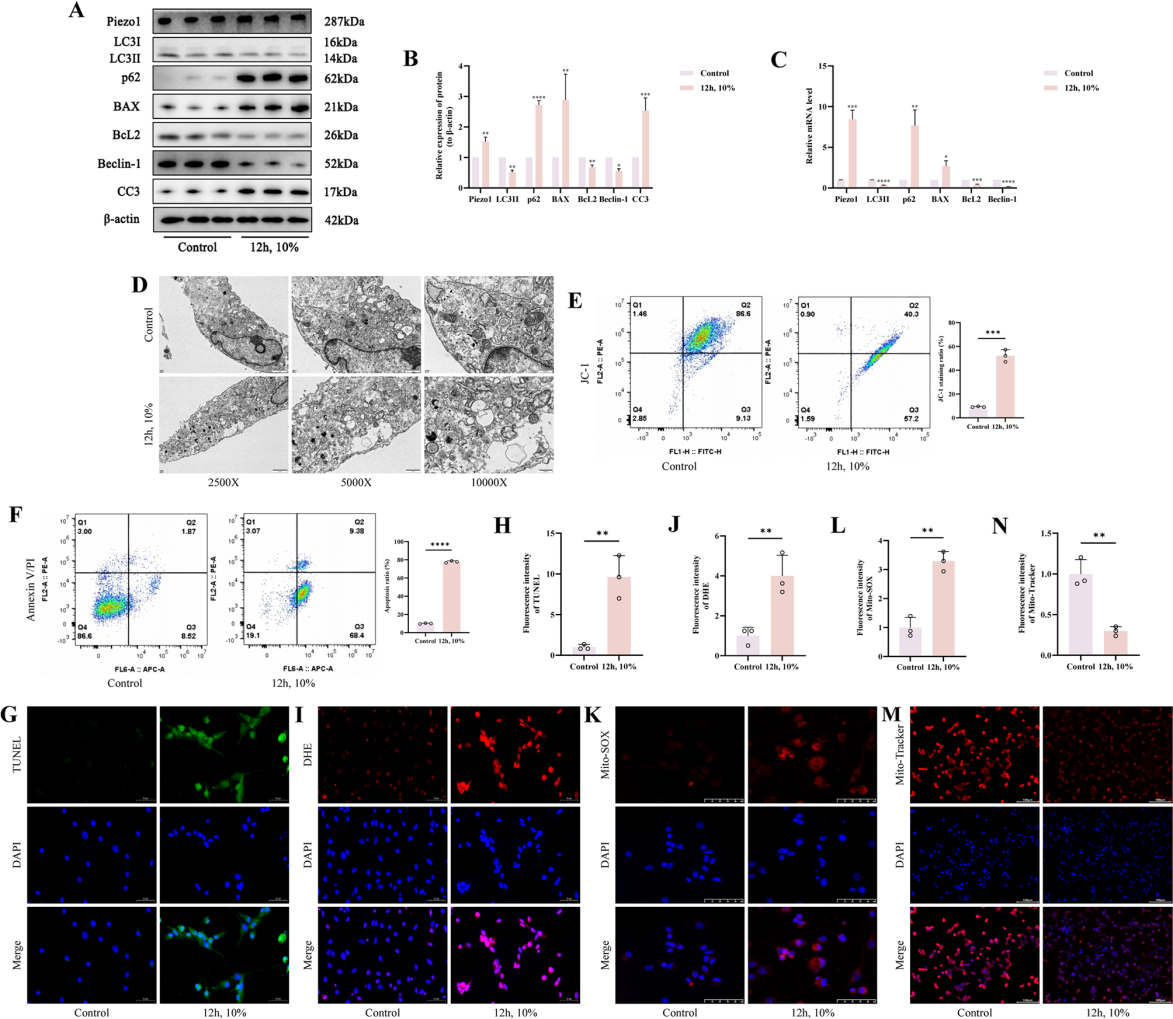
Mechanical Stress Overload Leads to Chondrocyte Mitochondrial Dysfunction, Apoptosis, and Autophagy Inhibition. **A**,** B** Western blot bands represent the expression levels of Piezo1, LC3, p62, BAX, BcL2, Beclin-1 and CC3 proteins in different groups of cartilage cells, with histograms representing the quantitative data of each index standardized to β-actin. **C** Detection of Piezo1, LC3, p62, BAX, BcL2 and Beclin-1 mRNA levels in cartilage cells by RT-qPCR. **D** Images of chondrocytes in different groups under TEM. **E** Flow cytometry plots and analysis bar graphs of different groups of cells detected by JC-1. **F** Flow cytometry plots and analysis bar graphs of different groups of cells detected by Annexin V-FITC/PI. **G**,** H** TUNEL staining fluorescence image and analysis bar graph of different groups of cells, accompanied by quantitative histograms. (Scale bar 50 μm). **I**,** J** DHE staining fluorescence image and analysis bar graph of different groups of cells, accompanied by quantitative histograms. (Scale bar 50 μm). **K**,** L** Mito-SOX Red staining fluorescence image and analysis bar graph of different groups of cells, accompanied by quantitative histograms. (Scale bar 50 μm). **M**,** N** Mito-Tracker Red staining fluorescence image and analysis bar graph of different groups of cells, accompanied by quantitative histograms. (Scale bar 100 μm) Values are expressed as the mean ± SD from three independent experiments (*n* = 3). *, **, *** and **** indicate *p* < 0.05, *p* < 0.01, *p* < 0.001 and *p* < 0.0001, respectively

To elucidate whether mechanical stress overload can affect mitochondrial function, apoptosis, and autophagy in chondrocytes, we conducted a series of in vitro validations. Initially, we discovered that the expression of proteins Piezo1, p62, BAX, and CC3 was significantly greater in the 12 h, 10% group as opposed to the Control group (*p* < 0.05), while the expression of LC3II, Bcl-2, and Beclin-1 was far lower (*p* < 0.05) (Fig. 3A, B). This trend was also reflected in the mRNA expression of the aforementioned markers (*p* < 0.05) (Fig. 3C). Subsequent observations with TEM of the intervened chondrocytes showed mitochondria in the Control group to be oval or round with intact membranes enclosed by a double membrane forming closed autophagosomes. In contrast, mitochondria in the 12 h, 10% group were incomplete with obvious swelling, cristae were disrupted or broken, appeared vacuolated, and there was a reduction in autophagosome numbers (Fig. 3D). Flow CytoMetry (FCM) assessment of mitochondrial membrane potential revealed that the JC-1 fluorescence intensity was considerably increased in the 12 h, 10% group, indicating a substantial decrease in mitochondrial membrane potential as opposed to the Control group (*p* < 0.05) (Fig. 3E). Similarly, Annexin V-FITC/PI FCM analysis indicated a far greater rate of apoptosis in the 12 h, 10% group than in the Control group (*p* < 0.05) (Fig. 3F). TUNEL fluorescent staining also suggested a significantly greater fluorescence intensity in the 12 h, 10% group relative to the Control group (*p* < 0.05) (Fig. 3G, H). DHE staining for cellular ROS indicated that the fluorescence intensity was significantly greater in the 12 h, 10% group than in the Control group (*p* < 0.05) (Fig. 3I, J). The fluorescence intensity of Mito-SOX Red was much greater in the 12 h, 10% group as opposed to the Control group (*p* < 0.05) (Fig. 3K, L), while the fluorescence intensity of Mito-Tracker Red was far lower than in the Control group (*p* < 0.05) (Fig. 3M, N). Therefore, we have determined that mechanical stress overload can promote apoptosis in chondrocytes, leading to mitochondrial dysfunction and inhibition of autophagy.

### Activation of Piezo1 exacerbates mechanically induced cell apoptosis and autophagy inhibition

**Fig. 4:**
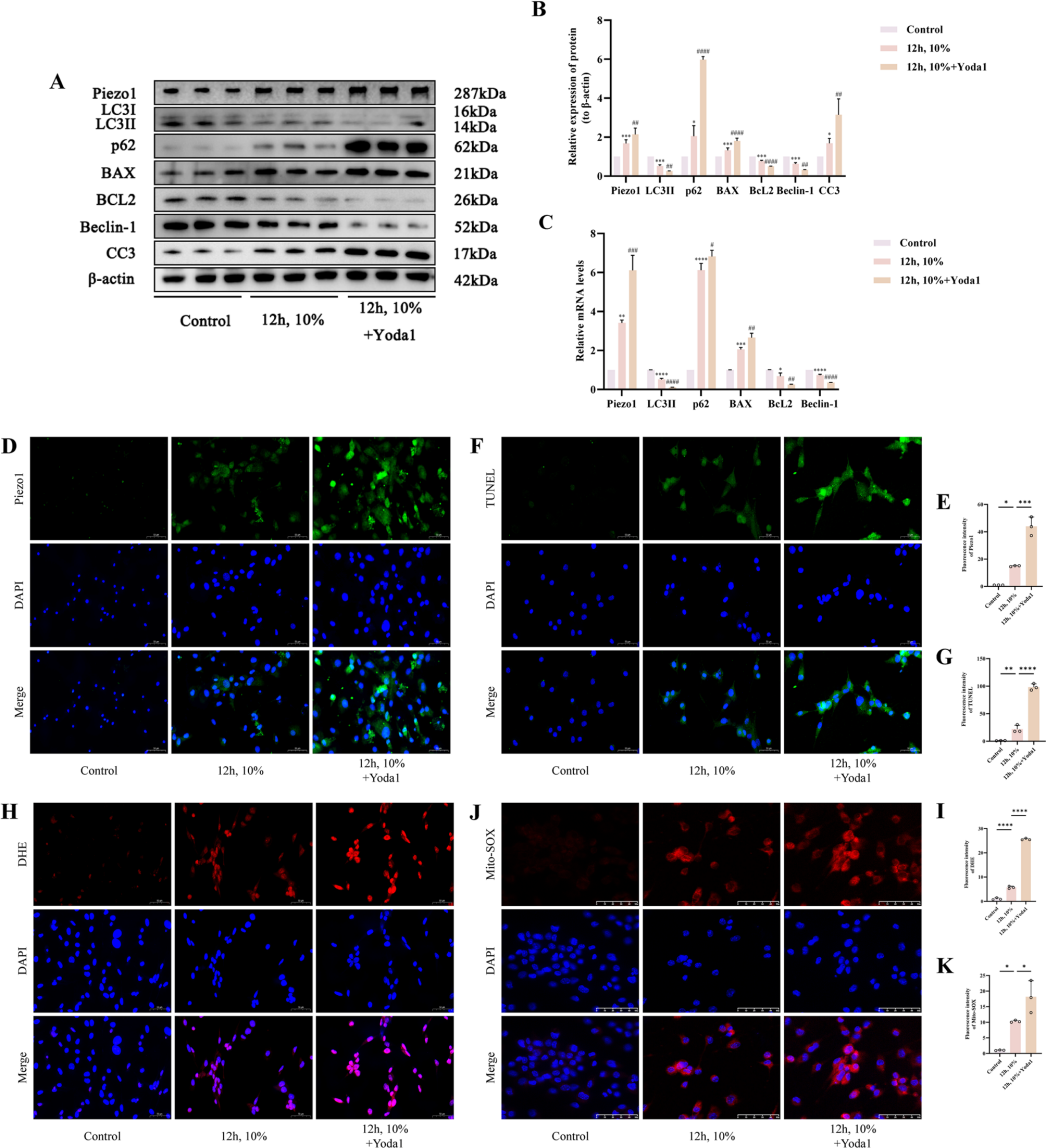
Activation of Piezo1 Exacerbates Mechanically Induced Cell Apoptosis and Autophagy Inhibition. **A**,** B** Western blot bands represent the expression levels of Piezo1, LC3, p62, BAX, BcL2, Beclin-1 and CC3 proteins in different groups of cartilage cells, with histograms representing the quantitative data of each index standardized to β-actin. **C** Detection of Piezo1, LC3, p62, BAX, BcL2 and Beclin-1 mRNA levels in cartilage cells by RT-qPCR. **D**,** E** Immunofluorescence detection of Piezo1 expression in different groups of cartilage cells, accompanied by quantitative histograms. (Scale bar 50 μm). **F**,** G** TUNEL staining fluorescence image and analysis bar graph of different groups of cells, accompanied by quantitative histograms. (Scale bar 50 μm). **H**,** I** DHE staining fluorescence image and analysis bar graph of different groups of cells, accompanied by quantitative histograms. (Scale bar 50 μm). **J**,** K** Mito-SOX Red staining fluorescence image and analysis bar graph of different groups of cells, accompanied by quantitative histograms. (Scale bar 50 μm) Values are expressed as the mean ± SD from three independent experiments (*n* = 3). *, **, *** and **** indicate *p* < 0.05, *p* < 0.01, *p* < 0.001 and *p* < 0.0001, respectively

To elucidate the role of Piezo1 activation in mechanically induced chondrocyte apoptosis and autophagy inhibition, we employed the Piezo1-specific agonist Yoda1 to intervene in cartilage cells. Initially, we observed that the protein expression of Piezo1, p62, BAX, and CC3 was far increased in the 12 h, 10%+Yoda1 group as opposed to the 12 h, 10% group (*p* < 0.05), while the expression of LC3II, Bcl-2, and Beclin-1 was considerably decreased (*p* < 0.05) (Fig. 4A, B). This trend was similarly reflected in the mRNA expression levels of the aforementioned markers (*p* < 0.05) (Fig. 4C). Subsequently, we noted a significant increase in the Piezo1 fluorescence intensity in Yoda1-treated chondrocytes in contrast to the untreated group (*p* < 0.05) (Fig. 4D, E). TUNEL fluorescence staining revealed that the fluorescence intensity in the 12 h, 10%+Yoda1 group was considerably greater than that in the 12 h, 10% group (*p* < 0.05) (Fig. 4F, G). DHE staining for cellular ROS fluorescence intensity also indicated a significant increase in the 12 h, 10%+Yoda1 group as opposed to the 12 h, 10% group (*p* < 0.05) (Fig. 4H, I). Additionally, the fluorescence intensity of Mito-SOX Red, indicative of mitochondrial oxidative stress, was significantly elevated in the 12 h, 10%+Yoda1 group in contrast to the 12 h, 10% group (*p* < 0.05) (Fig. 4J, K). Therefore, we infer that specific activation of Piezo1 promotes cell apoptosis and autophagy inhibition induced by mechanical stress overload.

### Mechanical stress overload-induced cell apoptosis and autophagy inhibition are associated with Piezo1 expression

**Fig. 5:**
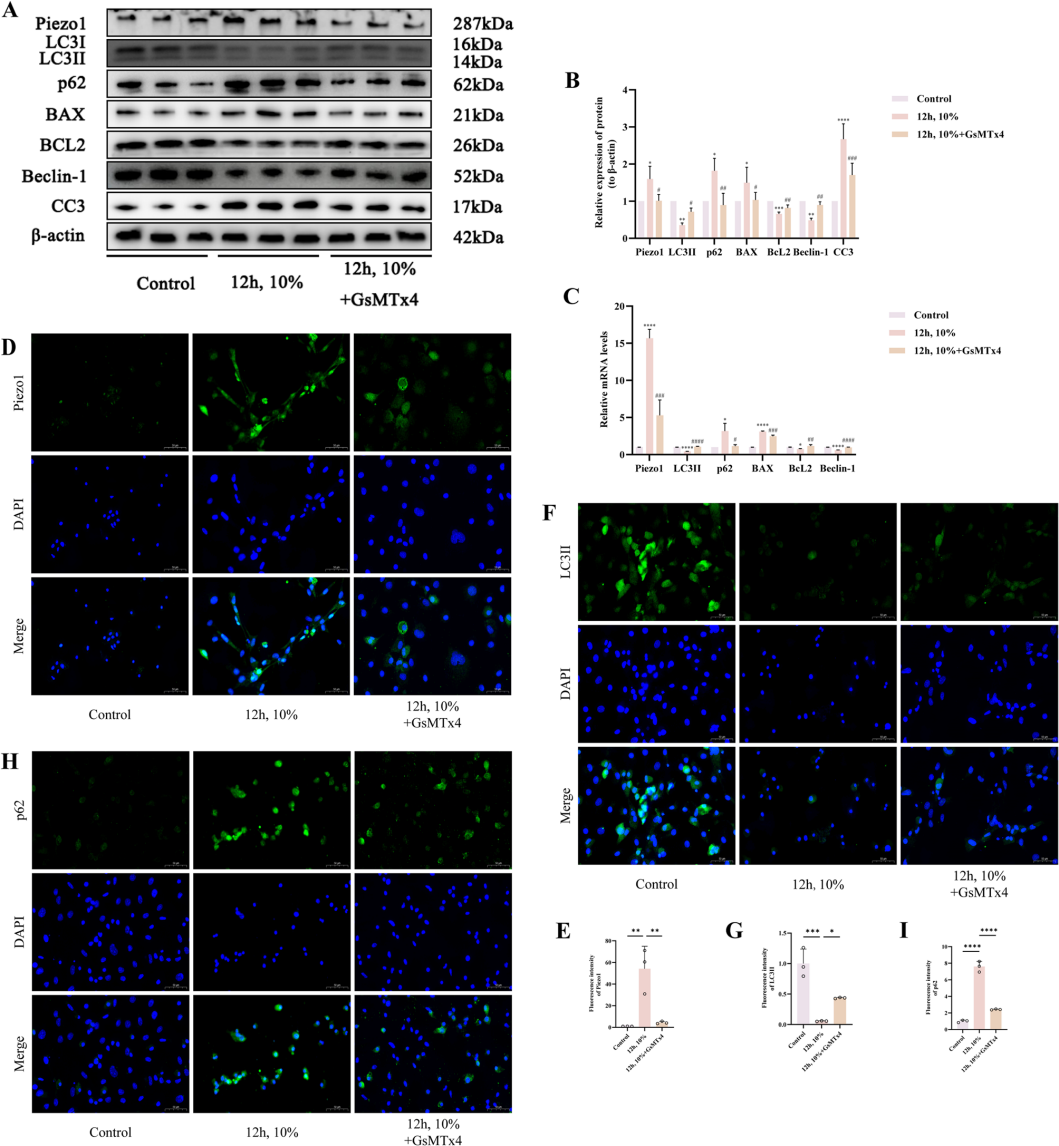
Mechanical Stress Overload-Induced Cell Apoptosis and Autophagy Inhibition Are Associated with Piezo1 Expression. **A**,** B** Western blot bands represent the expression levels of Piezo1, LC3, p62, BAX, BcL2, Beclin-1 and CC3 proteins in different groups of cartilage cells, with histograms representing the quantitative data of each index standardized to β-actin. **C** Detection of Piezo1, LC3, p62, BAX, BcL2 and Beclin-1 mRNA levels in cartilage cells by RT-qPCR. **D**,** E** Immunofluorescence detection of Piezo1 expression in different groups of cartilage cells, accompanied by quantitative histograms. (Scale bar 50 μm). **F**,** G** Immunofluorescence detection of LC3II expression in different groups of cartilage cells, accompanied by quantitative histograms. (Scale bar 50 μm). **H**,** I** Immunofluorescence detection of p62 expression in different groups of cartilage cells, accompanied by quantitative histograms. (Scale bar 50 μm) Values are expressed as the mean ± SD from three independent experiments (*n* = 3). *, **, *** and **** indicate *p* < 0.05, *p* < 0.01, *p* < 0.001 and *p* < 0.0001, respectively

To ascertain whether Piezo1 serves as a key target for mechanically stress overload-induced apoptosis and autophagy inhibition in chondrocytes, we utilized the Piezo1-specific inhibitor GsMTx4 to intervene in cartilage cell cultures. We initially observed that the protein expression levels of Piezo1, p62, BAX, and CC3 were considerably reduced in the 12 h, 10%+GsMTx4 group as opposed to the 12 h, 10% group (*p* < 0.05), while levels of LC3II, Bcl-2, and Beclin-1 were significantly elevated (*p* < 0.05) (Fig. 5A, B). This trend was similarly evidenced in the mRNA expression of the aforementioned markers (*p* < 0.05) (Fig. 5C). Subsequently, we noted a significant decrease in Piezo1 fluorescence intensity in GsMTx4-treated chondrocytes relative to the untreated group (*p* < 0.05) (Fig. 5D, E). Fluorescence staining for LC3II indicated a significant reduction in intensity in the 12 h, 10% group as opposed to the control group, while the intensity was far increased in the 12 h, 10%+GsMTx4 group in contrast with the 12 h, 10% group (*p* < 0.05) (Fig. 5F, G). Conversely, p62 fluorescence staining showed a marked increase in the 12 h, 10% group in contrast with the control group, whereas a significant decrease was observed in the 12 h, 10%+GsMTx4 group as opposed to the 12 h, 10% group (*p* < 0.05) (Fig. 5H, I). Hence, we propose that Piezo1 is among the critical targets for mechanical stress overload-induced apoptosis and autophagy inhibition in chondrocytes.

### Piezo1 mediates mitochondrial autophagy, intervening in mitochondrial dysfunction and cell apoptosis under stress stimulation

**Fig. 6:**
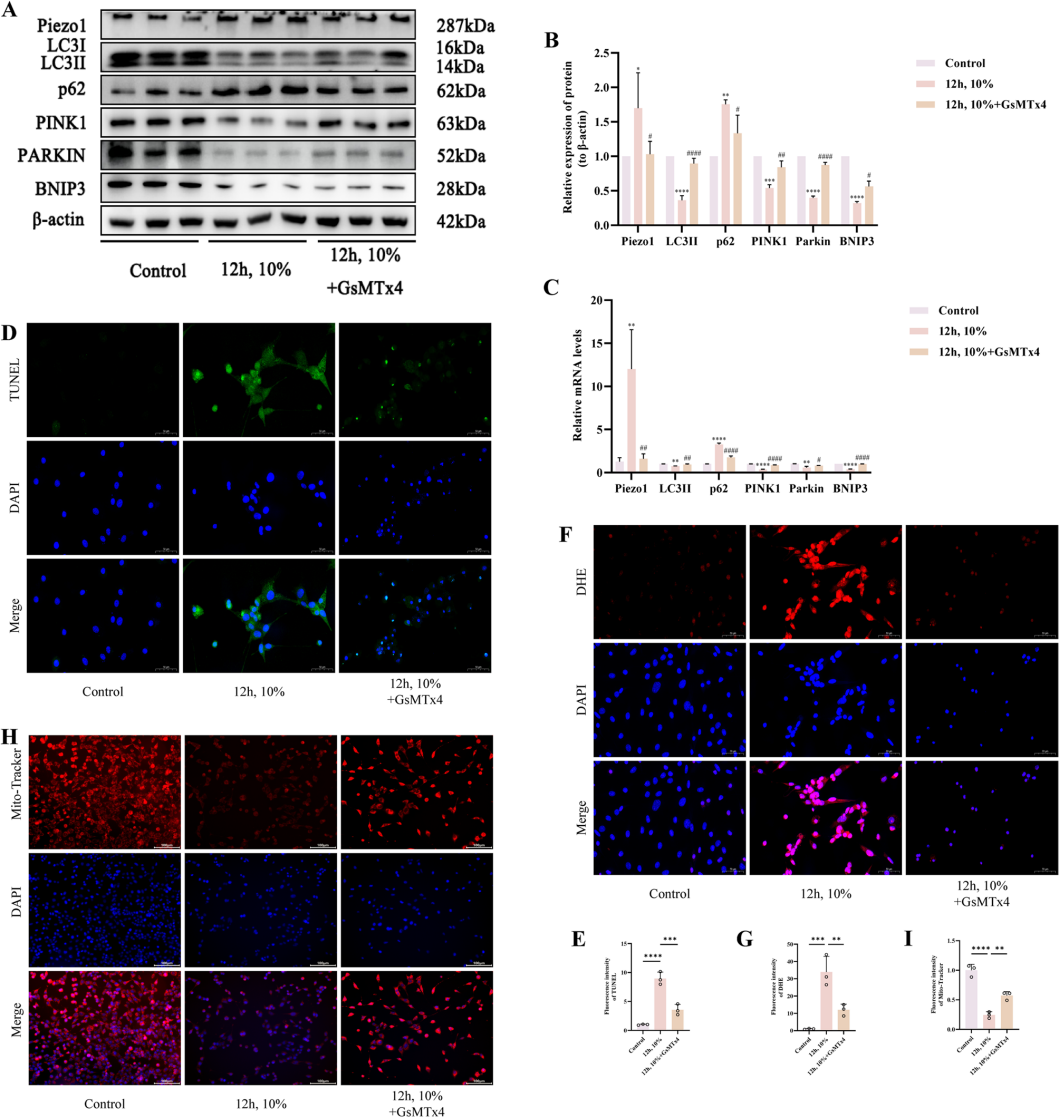
Piezo1 Mediates Mitochondrial Autophagy, Intervening in Mitochondrial Dysfunction and Cell Apoptosis Under Stress Stimulation. **A**,** B** Western blot bands represent the expression levels of Piezo1, LC3, p62, PINK1, Parkin and BNIP3 proteins in different groups of cartilage cells, with histograms representing the quantitative data of each index standardized to β-actin. **C** Detection of Piezo1, LC3, p62, PINK1, Parkin and BNIP3 mRNA levels in cartilage cells by RT-qPCR. **D**,** E** TUNEL staining fluorescence image and analysis bar graph of different groups of cells, accompanied by quantitative histograms. (Scale bar 50 μm). **F**,** G** DHE staining fluorescence image and analysis bar graph of different groups of cells, accompanied by quantitative histograms. (Scale bar 50 μm). **H**,** I** Mito-Tracker Red staining fluorescence image and analysis bar graph of different groups of cells, accompanied by quantitative histograms. (Scale bar 100 μm) Values are expressed as the mean ± SD from three independent experiments (*n* = 3). *, **, *** and **** indicate *p* < 0.05, *p* < 0.01, *p* < 0.001 and *p* < 0.0001, respectively

To further clarify the potential mechanisms by which mechanical stress overload induces chondrocyte apoptosis and autophagy inhibition via Piezo1, we validated these processes with the following in vitro experiments. Initially, we found that protein expression of Piezo1 and p62 was considerably increased in the 12 h, 10% group in contrast with the control group (*p* < 0.05), whereas the expression of LC3II, PINK1, Parkin, and BNIP3 was far decreased (*p* < 0.05). The use of GsMTx4 abrogated these trends (*p* < 0.05) (Fig. 6A, B). A similar pattern was observed for the mRNA expression of these markers (*p* < 0.05) (Fig. 6C). TUNEL fluorescence staining indicated a significant reduction in fluorescence intensity in the 12 h, 10%+GsMTx4 group as opposed to the 12 h, 10% group (*p* < 0.05) (Fig. 6D, E). DHE staining for cellular ROS fluorescence similarly showed a significant decrease in the 12 h, 10%+GsMTx4 group as opposed to the 12 h, 10% group (*p* < 0.05) (Fig. 6F, G). Finally, Mito-Tracker Red staining demonstrated that the fluorescence intensity was much greater in the 12 h, 10%+GsMTx4 group than in the 12 h, 10% group (*p* < 0.05) (Fig. 6H, I). Therefore, we propose that mechanical stress overload induces chondrocyte apoptosis and autophagy inhibition via Piezo1, and this process is closely related to mitochondrial function.

### Inhibition of Piezo1 expression attenuates mechanically overloaded-induced degeneration of knee cartilage

**Fig. 7:**
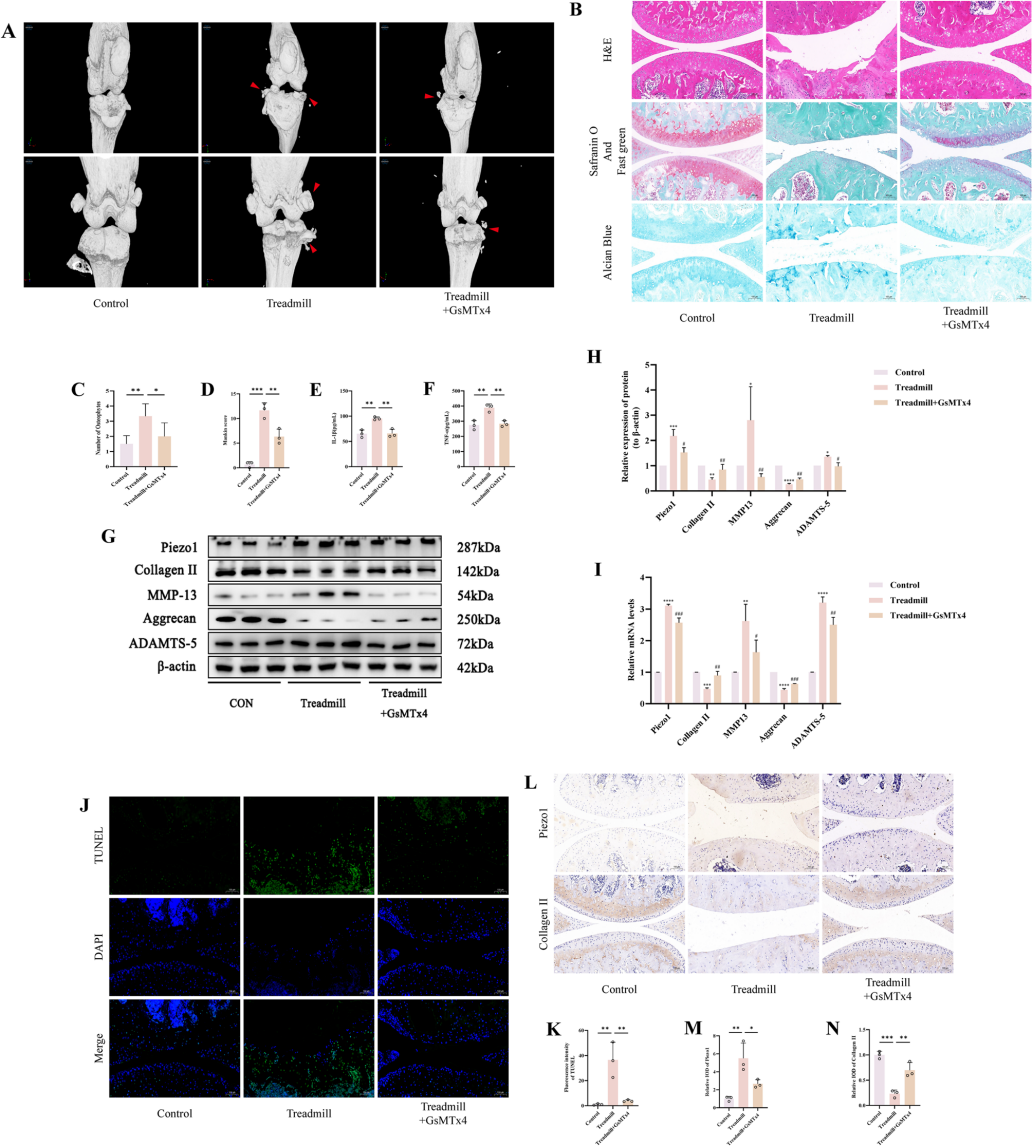
Inhibition of Piezo1 Expression Attenuates Mechanically Overloaded-Induced Degeneration of Knee Cartilage. **A** Micro CT images of knee joints of mice. Arrows show the formation of osteophytes. **B** Images of mouse cartilage stained with H&E, Safranin O-Fast Green and Alcian Blue. (Scale bar 100 μm) **C** Osteophyte number assay based on Micro-CT. **D** Mankin score of mouse knee joint. **E**,** F** Detection of IL-1β and TNF-α levels in mouse peripheral blood serum by ELISA. **G**,** H** Western blot bands represent the expression levels of Piezo1, Collagen II, MMP-13, Aggrecan and ADAMTS-5 proteins in different groups of cartilage tissue, with histograms representing the quantitative data of each index standardized to β-actin. **I** Detection of Piezo1, Collagen II, MMP-13, Aggrecan and ADAMTS-5 mRNA levels in cartilage tissue by RT-qPCR. **J**,** K** TUNEL staining fluorescence image and analysis bar graph of different groups of mouse caritage tissue, accompanied by quantitative histograms. (Scale bar 100 μm). **L-N** Piezo1 and Collagen II immunohistochemistry images of mouse cattilage tissue. (Scale bar 100 μm) Values are expressed as the mean ± SD from three independent experiments (*n* = 3). *, **, *** and **** indicate *p* < 0.05, *p* < 0.01, *p* < 0.001 and *p* < 0.0001, respectively

To elucidate whether the suppression of Piezo1 expression could ameliorate the cartilage damage induced by mechanical overload, we conducted a series of in vivo experiments for verification. Using Micro-CT analysis, we observed that the murine knee in the Treadmill + GsMTx4 group was presented fewer osteophytes compared to the Treadmill group (Fig. 7A, C). Follow-up histopathological staining with H&E, Saffron-O and Fast Green, and Alcian Blue indicated that the cartilage of the Treadmill + GsMTx4 group was more intact, displaying less wear and rupture, and a significant reduction in the Mankin score (*p* < 0.05) (Fig. 7B, D). ELISA assays of murine serum demonstrated that pro-inflammatory cytokines IL-1β and TNF-α were considerably decreased in the Treadmill + GsMTx4 group as opposed to the Treadmill group (*p* < 0.05) (Fig. 7E, F). WB analysis revealed that the expression of Collagen II and Aggrecan proteins significantly decreased in the Treadmill + GsMTx4 group as opposed to the Treadmill group (*p* < 0.05), while there was a huge increase in the expression of Piezo1, MMP-13, and ADAMTS-5 proteins (*p* < 0.05) (Fig. 7G, H). RT-qPCR analysis of the aforementioned markers corroborated these trends in mRNA expression (*p* < 0.05) (Fig. 7I). Whole-knee TUNEL fluorescence staining suggested a significantly greater fluorescence intensity in the Treadmill group as opposed to the Control group (*p* < 0.05), while the Treadmill + GsMTx4 group showed significantly lower intensity than the Treadmill group (*p* < 0.05) (Fig. 7J, K). IHC revealed that the expression of Piezo1 in the Treadmill + GsMTx4 group was significantly fewer than that in the Treadmill group (*p* < 0.05), the expression of Collagen II in the Treadmill group was far fewer than the Control group (*p* < 0.05), and the expression of Collagen II in the Treadmill + GsMTx4 group was significantly greater than in the Treadmill group (*p* < 0.05) (Fig. 7L, M, N). Therefore, our findings indicate that the inhibition of Piezo1 expression can improve knee cartilage damage resulting from mechanical stress overload.

## Discussion

Cartilage is a smooth connective tissue on the joint surface that reduces friction and dampens vibrations during knee movement, serving as a protective cushion (Natoli and Athanasiou 2009). Multiple factors may contribute to cartilage damage in KOA, including mechanical stress, inflammation, and metabolic changes (Diamond et al. 2024). The knee joint is the primary weight-bearing joint of the lower limbs. While regular exercise can protect cartilage tissue, excessive or abnormal stress stimulation can easily lead to chondrocyte death, increased matrix degradation, and altered cellular metabolism (Dwivedi et al. 2022). For example, during joint overuse, improper exercise, or joint mechanical imbalance, the articular cartilage layer is subjected to stress stimulation exceeding normal loads, leading to cartilage tissue damage and dysfunction (Atcha et al. 2021). Furthermore, a study comparing the effects of different exercise modalities on osteoarthritis demonstrated that, compared to swimming, treadmill exercise can achieve sufficient mechanical impact intensity to modulate anabolic signaling in the joint environment. Moderate-intensity treadmill exercise can even be utilized for constructing KOA models in Wistar rats (da Silva et al. 2023). Therefore, we established a KOA mouse model through treadmill exercise. Pathological sections of the knee joint revealed that excessive treadmill exercise-induced mechanical stress stimulation led to typical manifestations of knee cartilage damage, such as fissures, reduced thickness, and inflammatory factor infiltration. Subsequently, based on transcriptomic analysis combined with in vivo and in vitro experimental validation, this study found that abnormal mechanical stress triggers chondrocyte apoptosis and autophagy suppression, ultimately contributing to cartilage tissue damage—a phenomenon potentially linked to excessive Piezo1 activation.

In the context of bone and joint diseases, the molecular mechanisms mediated by Piezo1 are closely associated with mechanical stress stimulation. According to reports, Piezo1 acts as a regulatory factor in osteoblasts by sensing mechanical loads, affecting bone remodeling and mediating pathological new bone formation in ankylosing spondylitis through this mechanism (Chen et al. 2023). In cartilaginous tissue, Piezo1 can influence endochondral ossification and osteophyte formation during normal bone development (Brylka et al. 2024). Moreover, in experiments using destabilization of the medial meniscus surgery to impair mechanical stability of the mouse knee joint, researchers found that abnormal mechanical stress induced by joint instability leads to increased expression of Piezo1 in cartilage tissue, and knockdown of Piezo1 was shown to reduce cell death and exert a protective effect on the cartilage (Gan et al. 2024). However, the relationship between mechanical force intensity and Piezo1 expression remains incompletely understood. Therefore, in this study, a cell stretching apparatus was used to apply mechanical strain to primary chondrocytes, and the effects of different intensities and durations of mechanical stimulation were compared. We found that Piezo1 expression was significantly upregulated in chondrocytes subjected to 10% tensile strain for 12 h. Based on this result, mechanical stress was applied to chondrocytes, and subsequent in vitro experiments revealed that overexpression of Piezo1 modulated the expression of Bax, Bcl-2, and Beclin-1, as well as the LC3-II/I ratio, thereby influencing chondrocyte viability. Specifically, Piezo1 overexpression exacerbated stress-induced apoptosis and autophagy inhibition. These findings suggest that Piezo1 may serve as an intermediary between mechanical stimulation and cell death, which is consistent with previous studies showing that inhibition of Piezo1 can attenuate mechanical stress-induced chondrocyte apoptosis and delay cartilage degeneration in the progression of KOA (Sun et al. 2021a, b). To elucidate the specific role of Piezo1 in chondrocytes, our study employed the selective inhibitor GsMTx4 to block Piezo1 activation during mechanical stress loading. Previous studies have reported that GsMTx4 can protect chondrocytes from explant-induced cell death by inhibiting the expression of Piezo1 (Lee et al. 2014). Our results indicate that GsMTx4 reduces apoptosis and promotes autophagy in chondrocytes under mechanical stress, thereby enhancing cell survival. These findings suggest that the mechanotransduction pathways mediating chondrocyte apoptosis and autophagy may be dependent on Piezo1 expression.

During the application of tensile stress to chondrocytes, we observed an increase in mitochondrial ROS production and alterations in mitochondrial membrane potential. Subsequent transmission electron microscopy revealed morphological alterations in mitochondria of the stress-stimulated chondrocytes, along with a relative reduction in autophagosome numbers, suggesting a potential link to subsequent events such as chondrocyte apoptosis. Autophagy is a biological process of self-digestion and recycling by the cell, primarily degrading harmful or unnecessary components into basic molecules via lysosomes, thus maintaining intracellular equilibrium and contributing to the preservation of normal cellular function and deceleration of the aging process (Araya et al. 2019; Glick et al. 2010). These findings suggest that autophagy may serve as a homeostatic regulatory mechanism in cartilage, aiding chondrocyte survival under adverse conditions (Kong et al. 2024). To investigate the potential mechanism by which Piezo1 influences chondrocyte autophagy under mechanical stress, we examined changes in the expression of classical autophagy-related signaling pathways following GsMTx4 treatment. PINK1, a serine/threonine kinase targeted to mitochondria, accumulates on the outer membrane of damaged mitochondria. Subsequently, phosphorylated E3 ubiquitin ligase—PARKIN facilitates the translocation of damaged mitochondria to nascent autophagosomes, enabling their clearance through the autophagic pathway (Zimmermann and Reichert 2017). In renal diseases, PINK1/Parkin-mediated mitophagy has been shown to prevent cisplatin-induced apoptosis of renal tubular epithelial cells and tissue damage both in vivo and in vitro (Wang et al. 2018). Additionally, the Parkin-independent pathway of mitophagy is mediated by BNIP3, which acts by directly binding to Atg8 family proteins, predominantly responsible for mitochondrial autophagy under hypoxic conditions (Yuan et al. 2017). Consistent with previous studies, our findings demonstrate that GsMTx4 upregulates the expression of suppressed PINK1, Parkin, and BNIP3 in chondrocytes, while also alleviating mitochondrial damage and apoptosis. These results suggest that reducing Piezo1 activation may improve mitochondrial autophagy impairment under mechanical stress and mitigate cartilage damage.

Finally, we conducted in vivo experiments on a treadmill exercise-induced KOA mouse model to examine the protective effects of Piezo1 inhibition on cartilage tissue. The results showed that GsMTx4 treatment in KOA mice led to reduced peripheral blood circulation inflammation, improved cartilage erosion and damage, and decreased chondrocyte apoptosis. These findings confirm that downregulation of Piezo1 can suppress apoptosis induced by excessive mechanical stress, thereby delaying the pathological progression of KOA. However, this study has certain limitations. First, we only observed the inhibitory effects of Piezo1 activation on autophagy and mitophagy, and the specific mechanisms of Piezo1-mediated PINK1/Parkin and BNIP3 pathways require further investigation. Secondly, Piezo1 is a cation channel that responds to various mechanical forces, including shear stress, osmotic pressure, and tensile deformation (Lei et al. 2024). In experiments related to chondrocytes, researchers often use systems such as the Flexcell tension system or multi-channel stretching stress loading systems to apply continuous tensile forces to cells, thereby activating mechanosensitive channels like Piezo1 (Ren et al. 2023; Sun et al. 2020). However, excessive exercise or joint mechanical instability applies complex mechanical stress to cartilage tissue. We cannot generalize the effects of all types of mechanical stress, as tensile and compressive forces may differ in their activation of Piezo1 and potentially have distinct effects on cartilage tissue. Therefore, we hope to conduct more detailed studies on this issue in future experiments.

**Fig. 8:**
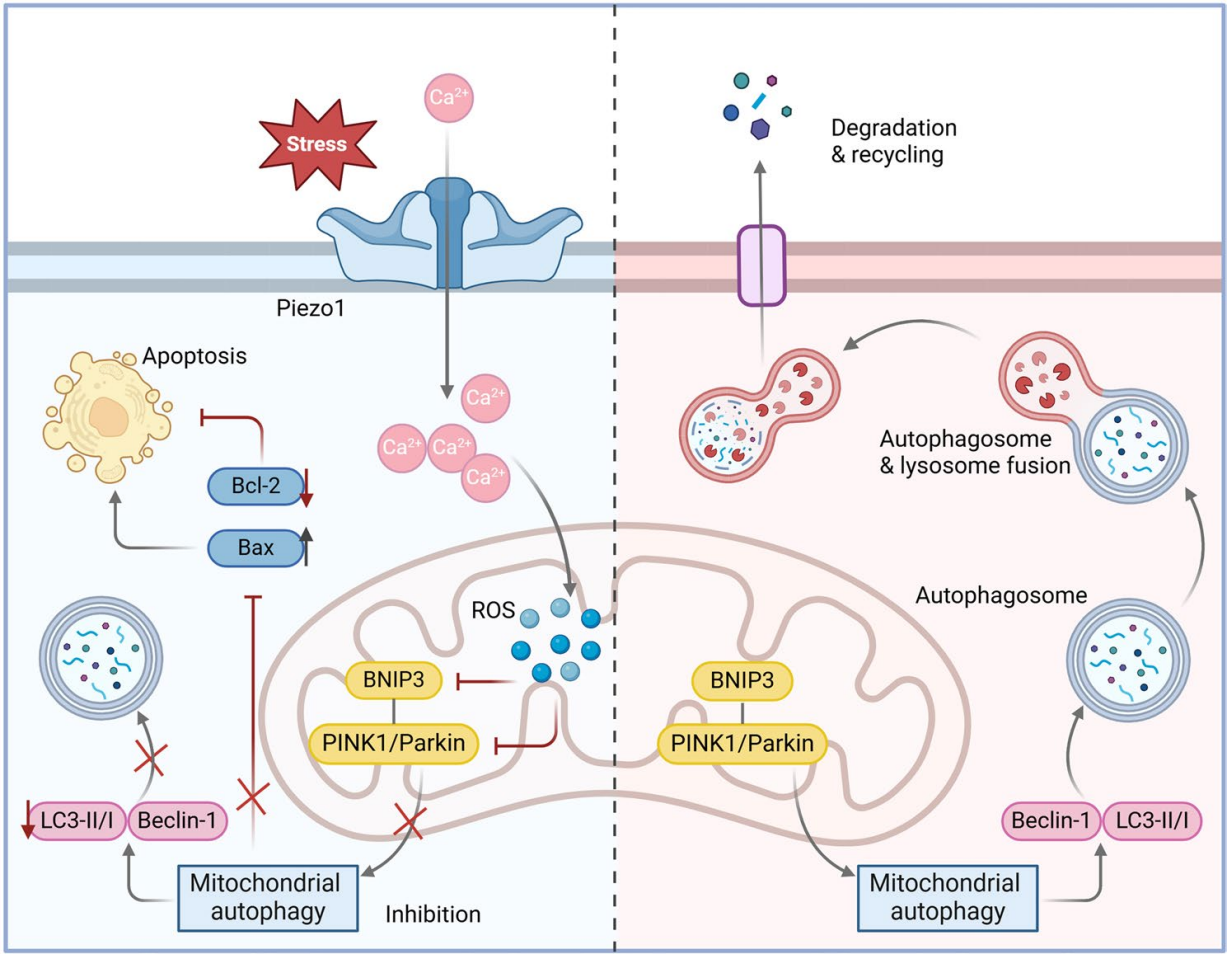
Mechanism schematic diagram of this study

In conclusion, excessive mechanical stress loading may activate the Piezo1 channel, leading to autophagy impairment and apoptotic damage in chondrocytes, ultimately exacerbating the progression of KOA (Fig. 8).

## Supplementary Information

Supplementary Material 1.

## Data Availability

No datasets were generated or analysed during the current study.

## Abbreviations

OA: Osteoarthritis
KOA: Knee osteoarthritis
ECM: Extracellular matrix
LC3: Microtubule-associated protein 1 light chain 3
ROS: Reactive oxygen species
H&E: Hematoxylin-Eosin
IL-1β: Interleukin-1β
TNF-α: Tumor necrosis factor-α
ELISA: Enzyme linked immunosorbent assay
MMP-13: Matrix metalloproteinases-13
ADAMTS-5: Recombinant A Disintegrin and Metalloproteinase with Thrombospondin 5
BCL2: B-cell lymphoma-2
Bax: BCL-2-associated X protein
PINK1: PTEN induced putative kinase 1
BNIP3: BCL2 interacting protein 3
RT-qPCR: Real-time quantitative PCR
PFA: Perfluoroalkoxy
IOD: Integrated optical density
SD: Standard deviation
ANOVA: Analysis of variance
KEGG: Kyoto Encyclopedia of Genes and Genomes
PCA: Principal Components Analysis
IHC: Immunohistochemical
WB: Western blotting
CC3: Cleaved caspase-3
TEM: Transmission Electron Microscopy
FCM: Flow CytoMetry
DHE: Dihydroethidium
