# Supplementary material for: Piezo1 induces mitochondrial autophagy dysfunction leading to cartilage injury in knee osteoarthritis
Source: Mol Med. 2025 Aug 2;31:272. doi: 10.1186/s10020-025-01335-x (PMC12317449; doi:10.1186/s10020-025-01335-x)
Supplement: Supplementary file 1 — Supplementary Material 1. [file 10020_2025_1335_MOESM1_ESM.docx]

**1. Transfection**

Chondrocyte were transfected with siPiezo1 (GeneID: 361430) using Lipofectamine 3000, along with a three-plasmid system and a Negative Control. The successful knockdown of Piezo1 was confirmed by assessing the protein level through RT-qPCR analysis (Table S1 and Fig. S1).

| Name | Sequence (5'-3') | |
| --- | --- | --- |
|  | Sense | Antisense |
| siRNA-Piezo1-1 | GCAUCAAGUUCAUCGACUATT | UAGUCGAUGAACUUGAUGCTT |
| **siRNA-Piezo1-2** | **CCUGGUACAUGUACCAUGAATT** | **UUCAUGGUACAUGUACCAGGTT** |
| siRNA-Piezo1-3 | GGACAAUCUUCAGGUACAATT | UUGUACCUGAAGAUUGUCCTT |

**Table S1 Sequences of siRNA-Piezo1**


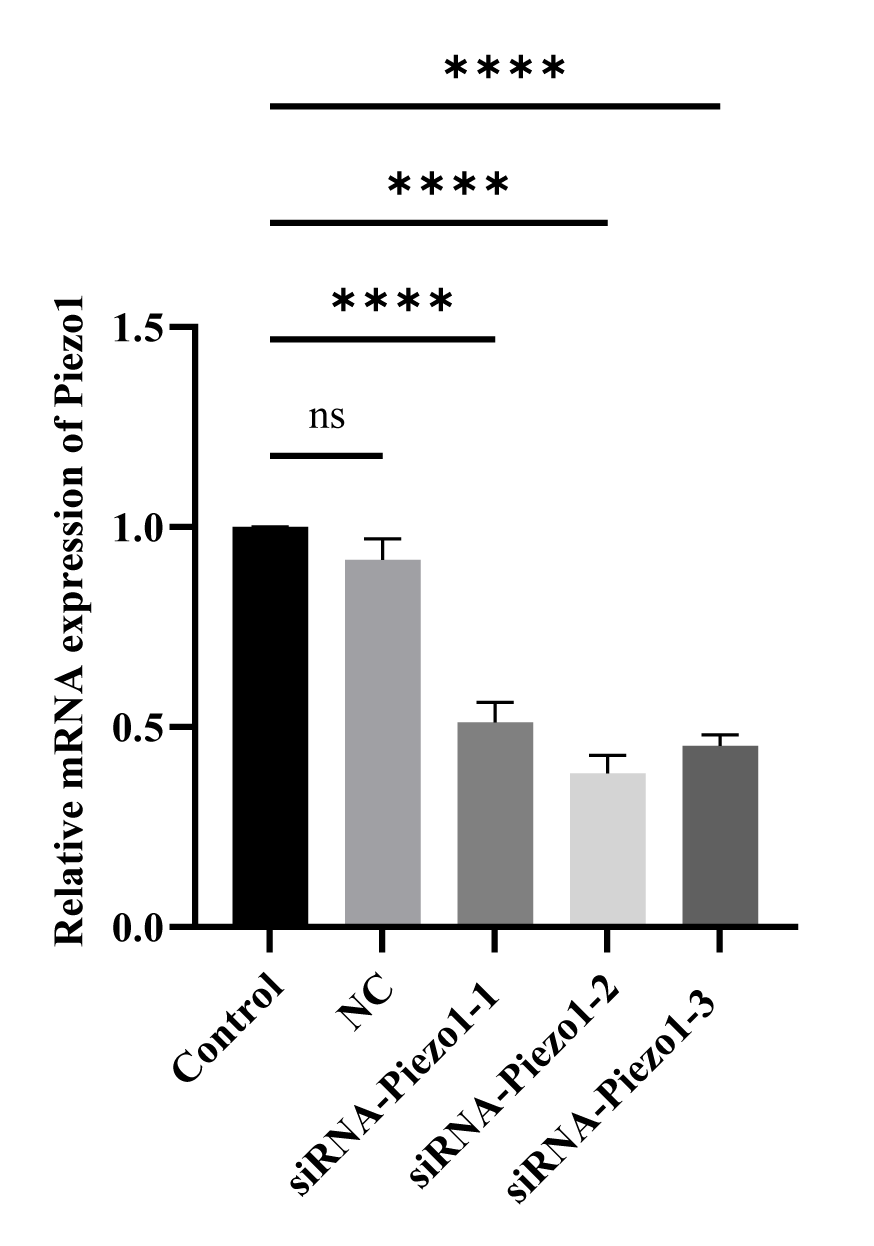


**Fig. S1 RT-qPCR was used to detect the Piezo1 mRNA level, validating the success of knocking down Piezo1 in chondrocytes. siRNA-Piezo1-2 (si-Piezo1) was the screening target in this study.**

All data are presented as mean ± standard deviation (n = 3). *****P* < 0.001.


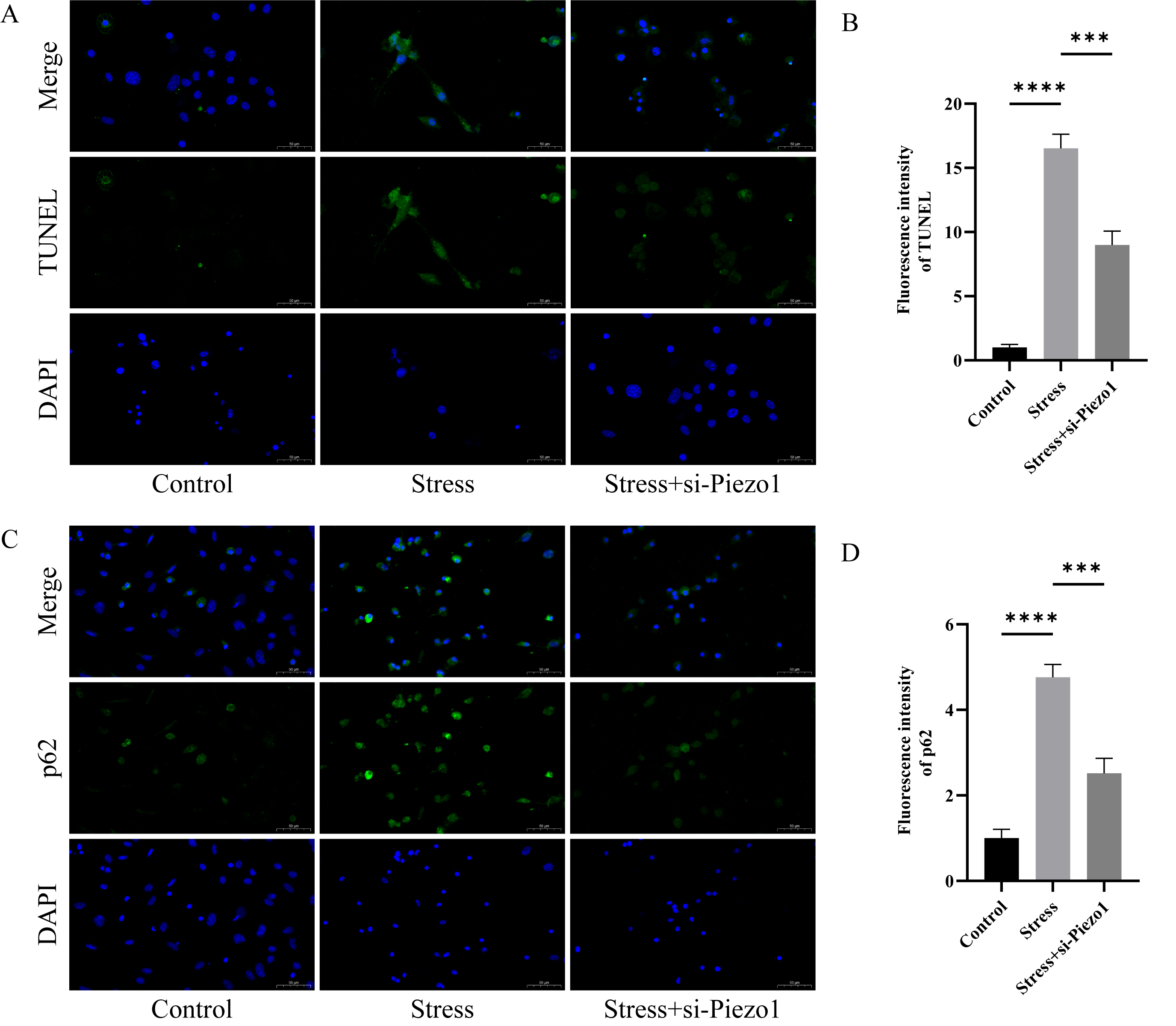


**Fig. S2 Immunofluorescence of chondrocytes after si-Piezo1 intervention.**

**A, B** TUNEL staining fluorescence image and analysis bar graph of different groups of cells, accompanied by quantitative histograms. (Scale bar 50 μm)

**C, D** Immunofluorescence detection of p62 expression in different groups of cartilage cells, accompanied by quantitative histograms. (Scale bar 50 μm)

All data are presented as mean ± standard deviation (n = 3). *****P* < 0.001, ****P* < 0.001.
